# Editor’s Note: Relates to: ‘Immuno-antibiotics: targeting microbial metabolic pathways sensed by unconventional T cells’

**DOI:** 10.1093/immadv/ltab023

**Published:** 2021-11-19

**Authors:** Matthias Eberl, Eric Oldfield, Thomas Herrmann

**Affiliations:** 1 Division of Infection and Immunity, School of Medicine, Cardiff University, Cardiff, UK; 2 Systems Immunity Research Institute, Cardiff University, Cardiff, UK; 3 Department of Chemistry, University of Illinois at UrbanaChampaign, Urbana, IL, USA; 4 Institut für Virologie und Immunbiologie, Julius-Maximilians-Universität Würzburg, Würzburg, Germany

We would like to alert readers to the recent retraction of one of the papers (Singh *et al*. [ref. 22]) cited in this review, which advocated the use of IspH inhibitors as ‘immuno-antibiotics’. The authors of the review discuss some of the findings by Singh *et al*. in the text and make reference to the work in Table 1 and Figure 2. Despite the retraction of this paper from the literature, the Editors at Immunotherapy Advances agree with Eberl *et al*. that there is a large body of complementary evidence in the literature demonstrating that the MEP pathway is an attractive target for the development of novel antibiotics and that manipulation of the MEP pathway has a direct effect on anti-microbial γδ T cell responses. As such we are confident that this retraction does not affect the validity of their article.

